# Amtolmetin: A Reappraisal of NSAID with Gastroprotection

**DOI:** 10.1155/2016/7103705

**Published:** 2016-03-22

**Authors:** Amit Garg, Ahsan Shoeb, Latha Subramanya Moodahadu, Akhilesh Sharma, Arul Gandhi, Shyam Akku

**Affiliations:** Global Medical Affairs, Dr. Reddy's Laboratories Ltd., Hyderabad 500034, India

## Abstract

*Aim*. To assess the gastrosparing effect of amtolmetin guacyl (AMG) against other nonsteroidal anti-inflammatory drugs (NSAIDs) in patients with osteo-/rheumatoid arthritis.* Methods.* A literature search was done in the electronic databases (PubMed, Google Scholar, Embase, and Scopus) with key words “amtolmetin guacyl”, “amtolmetin”, and “arthritis”; filters were applied to obtain publications between 01-Jan-1985 and 01-Oct-2015, which were “clinical trials” in osteo-/rheumatoid arthritis patients and in “English language.” Studies were assessed using the Jadad criteria and trials with score ≥ 3 were included in the analysis to compare the safety and efficacy of AMG against other NSAIDs.* Results.* Search yielded 19 publications of which 3 were included for analysis. Baseline characteristics of patients were comparable between the AMG group and other NSAIDs (diclofenac, celecoxib, and piroxicam) groups in all trials. Efficacy of AMG was similar to the other NSAIDs compared in the trials. The number of adverse events (AEs) reported was similar between both the groups; however, severe AEs reported were significantly lower in the AMG group. Of note was the significant lower number of duodenal ulcers after treatment in the AMG group.* Conclusions.* AMG has efficacy similar to other NSAIDs and a safer gastrointestinal AE profile when compared to the other NSAIDs.

## 1. Introduction

Nonsteroidal anti-inflammatory drugs (NSAIDs) are extensively used in the treatment of chronic painful conditions such as rheumatoid arthritis (RA) and osteoarthritis (OA) to alleviate pain and inflammation associated with the disease and have become the mainstay of therapy in these conditions [1, 2]. Major limiting factor in long-term use of NSAIDs is their safety, mainly gastrointestinal (GI) adverse effects ranging from mild to severe dyspeptic symptoms to the development of gastric or duodenal ulceration, hemorrhage, or perforation, which adversely affect patient's quality of life [1–3]. These complications indicate a clear unmet need in the safety of current treatment options for the management of RA and OA. NSAIDs which are more gastric tolerable are to be preferred for long-term use in these conditions. One such compound is amtolmetin guacyl (2-methoxyphenyl-1-methyl-5-p-methylbenzoyl-pyrrole-2-acetamido acetate, AMG) derived from the fusion between tolmetin with guaiacol and glycine, which is a nonselective NSAID having a cyclooxygenase-2/cyclooxygenase-1 (COX-2/COX-1) selectivity ratio of 4.4 [4].

Amtolmetin guacyl (AMG) was demonstrated to be an effective anti-inflammatory drug with better GI tolerability profile displaying substantially lower incidence of GI adverse events compared to traditional NSAIDs [5, 6]. This gastric sparing effect of AMG has been attributed to the presence of vanillic moiety in the molecule, which stimulates capsaicin receptors and releases calcitonin gene related peptide (CGRP) and consequently increases nitric oxide (NO) production [7], hence counterbalancing the deleterious effects of prostaglandin depletion due to COX inhibition and providing mucosal protection. Despite these favorable characteristics, its use has been limited owing to availability of newer NSAIDs. We analyzed earlier studies and compared the GI sparing effect of AMG with other widely used NSAIDs in patients with OA and RA to offer pragmatic suggestions for clinical practice.

## 2. Methods

A literature search was done in the electronic databases (PubMed, Google Scholar, Embase, and Scopus) with the following key words: “amtolmetin guacyl”, “amtolmetin”, and “arthritis”; appropriate filters were applied in order to obtain publications between 01-Jan-1985 and 01-Oct-2015, which were “clinical trials,” conducted in patients with osteo-/rheumatoid arthritis and in “English language.”

**Figure 1:**
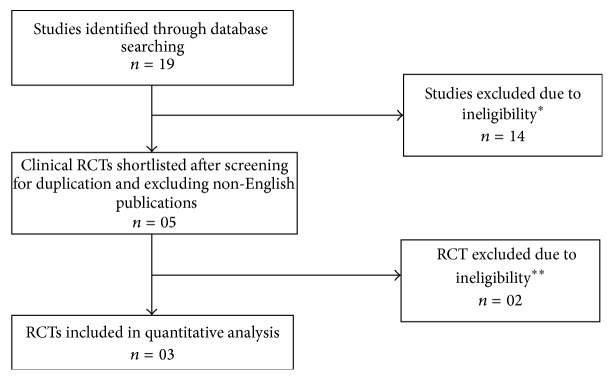
Literature search results.   ^*∗*^Preclinical studies, nonrandomized trials.   ^*∗∗*^Jadad score 2, no significant gastrointestinal components discussed.

Search result is shown in Figure 1. The quality of each randomized clinical trial was assessed using the Jadad criteria and trials with score ≥ 3 were considered for the analysis [23]. Jadad score is a five-point score (1: low; 5: high quality) which was developed by Jadad AR in 1996 and is well validated for trials involving pain therapy. The Jadad criteria are simple and easy to use criteria that incorporate the most important individual components of methodological quality which includes randomization, blinding, and handling of patient attrition. However, it is limited by placing greater emphasis on the quality of reporting as opposed to the actual methodological quality of a trial. Olivo et al. [24] found that the Jadad scale demonstrated the strong evidence in terms of validity and reliability. Results from these shortlisted trials were computed to compare the safety of AMG against various other NSAIDs, with a focus on the GI adverse events.

### 2.1. Statistical Analysis

Data was captured in Microsoft Office Excel worksheet. Descriptive analysis was performed for all demographic variables. Student's *t*-test, Chi-square test, and post hoc test were used to test the hypothesis. The statistical tests were two-sided at the 5% level of significance. Statistical analysis was performed by using STATA version 13 for Windows (Stata Corp., College Station, TX).

## 3. Results

Initial search result yielded 19 publications of which only 4 met our selection criteria (Table 1). Of the 4 studies, 3 studies were considered for analysis and one study was excluded as gastrointestinal adverse effects were not discussed. The baseline characteristics of patients were comparable between the AMG and the other NSAIDs (Diclofenac, Celecoxib, and Piroxicam) groups in all these trials (Table 2). Efficacy of AMG was similar to the other NSAIDs compared in these trials. The numbers of adverse events (AEs) recorded was similar between both groups (Table 3); however, severe adverse events reported were significantly lower in the AMG group. Of note was the significant lower number of duodenal ulcers after treatment in the AMG group.

From these selected studies, the number of patients evaluated was 166 (48.3%) in AMG group and 177 (51.6%) in other NSAIDs' group. These patients were diagnosed to have either OA or RA. Females outnumbered men in both groups, 137 versus 29 (82.5% versus 17.47%) in AMG group and 141 versus 36 (79.7% versus 20.3%) in other NSAIDs' group. Mean age (±SD) of patients was 58.97 (±3.2) in AMG group and 57.83 (±3.0) years in other NSAIDs' group.

Overall, gastrointestinal symptoms were seen in 80 (48.2%) and 91 (51.4%) patients in AMG and other NSAIDs' group, respectively. Table 3 shows GI symptoms reported from these three studies. There was no statistically significant difference in the occurrence of gastric symptoms in these two groups, but a significant lower mean percentage of severe gastric and/duodenal ulcer after treatment was observed with AMG. Withdrawal from the study due AEs was 5 (3%) in AMG and 15 (8.5%) in other NSAIDs' group, which was statistically significant (*p* < 0.05). There was a significant difference in mean percentage of serious AE leading to withdrawal from the study between these two groups favoring the AMG group. We also noted that there was a statistically significant difference in mean percentage of high endoscopy score between AMG (21.4%) and other NSAIDs (27.6%, *p* < 0.05).

## 4. Discussion

NSAIDs are among the most commonly used drugs in the world. In Europe, NSAIDs represent more than 7.7% of all prescriptions and in 2004 a total of 111 million NSAID prescriptions were written in the United States alone [1, 2]. Due to the noticeable efficacy and affordability, majority of patients with chronic painful conditions such as RA and OA are managed by NSAIDs. Gastric intolerability is the most important and frequent adverse event and disadvantage of the current NSAIDs which may compel the patient to discontinue therapy [3]. With long-term usage of NSAIDs, development of GI related morbidities is apparent. Nearly 25% patients develop endoscopically visible ulcers after 3 months of NSAID usage and nearly 10% of patients in NSAID clinical trials withdraw from the trials because of adverse events [25, 26]. Hence, in clinical practice, gastric acid neutralizers are coprescribed along with NSAIDs to manage GI adverse effects and for better patient compliance which is an additional financial burden for these patients. There is an unmet medical need for analgesics that have a better safety profile and can be used on a long-term basis for chronic pain management. Thus, it would be appropriate to identify an analgesic and anti-inflammatory compound with better gastric tolerability.

Amtolmetin has demonstrated efficacy similar to other NSAIDs with a better gastric sparing effect in various studies. Unlike other NSAIDs, AMG is preferentially administered on an empty stomach, as the maximum activation of gastric capsaicin receptors takes place on empty stomach. We identified studies that evaluated AMG and shortlisted these based on the Jadad score to compare the GI tolerability with other NSAIDs in patients with RA or OA.

Demographically, female patients were more in these studies analyzed. Our analysis of these selected studies supports the gastric sparing effect of AMG, which is further proved by endoscopic findings in two studies. Significantly high endoscopy score was seen in other NSAIDs' group (27.6%) compared to AMG (21.4%; *p* < 0.05). Though the number of AEs was similar among both the groups, the number of patients with severe gastric and/or duodenal ulcer after treatment was significantly more in other NSAIDs' group (14.2%) compared to AMG (4.3%; *p* < 0.05).

Withdrawals due to GI adverse effects were more common in other NSAIDs' group than in AMG, indicating that the latter can contribute to better patient compliance. This in turn may result in improved symptom-free intervals, thus yielding better quality of life and productivity.

Literature and results of our analysis suggest that AMG's gastric sparing effect can overcome the limitation associated with long-term use of NSAID especially in painful chronic inflammatory disorders. With the increasing GI safety concerns with other NSAIDs, AMG seems to be a feasible treatment option.

Though AMG has been approved for the treatment of painful disorders in Italy (2008), Russia (2014), and India (2008), there is paucity of data and it is underutilized in clinical practice. Long-term studies on large number of patients can provide further data on safety and its effect on quality of life in patients with painful chronic inflammatory conditions.

## 5. Conclusions

Amtolmetin guacyl with its established efficacy that is comparable to routinely prescribed NSAIDs is worth considering for chronic pain management in patients with osteoarthritis or rheumatoid arthritis. It has a good safety profile, particularly with its gastrointestinal sparing effect that can improve treatment compliance in these patients.

## Figures and Tables

**Table 1:** List of all studies considered and excluded with Jadad scores and reasons for exclusion.

Serial number	Reference	Jadad score	Reason for exclusion
1	Jajić et al. [4]	3	—
2	Montrone et al. [6]	3	—
3	Bianchi Porro et al. [8]	3	—
4	Niccoli et al. [9]	3	No significant gastrointestinal components discussed
5	Lazzaroni et al. [5]	2	Study conducted in healthy volunteers
6	Kirkova et al. [10]	—	In vivo study
7	Sostres et al. [11]	—	Not a RCT
8	Pisano et al. [7]	—	Preclinical study
9	Coruzzi et al. [12]	—	Preclinical study
10	Tubaro et al. [13]	—	Preclinical study
11	Tubaro et al. [14]	—	Preclinical study
12	Patrignani et al. [15]	—	Not a RCT
13	Li et al. [16]	—	Preclinical study
14	Rong et al. [17]	—	Preclinical study
15	Allison et al. [18]	—	Not a RCT
16	Riezzo et al. [19]	—	Study in healthy volunteers; GI adverse effects not discussed
17	Vicari et al. [20]	—	Not a RCT, published in Italian
18	Morini et al. [21]	—	Preclinical study
19	Hotha et al. [22]	—	Preclinical study

**Table 2:** Demographic profile of the patients from shortlisted studies.

	Bianchi Porro et al. [8] (4 weeks)		Montrone et al. [6] (30 days)		Jajić et al. [4] (24 weeks)		Age (year) mean ± SD	
	AMG	Diclofenac	AMG	Piroxicam	AMG	Celecoxib	AMG	NSAIDs
Patients evaluated, *n*	32	32	49	50	85	95	58.0 ± 3.2	57.8 ± 3.0
Male, *n* (%)	6 (18.8)	5 (15.6)	5 (10.2)	10 (20)	18 (21.2)	21 (22.1)		
Female, *n* (%)	26 (81.2)	27 (84.4)	44 (89.8)	40 (80)	67 (78.8)	74 (77.9)		

**Table 3:** Comparisons of adverse events in different trials.

	Bianchi Porro et al. [8]		Montrone et al. [6]		Jajić et al. [4]		*p* value
	AMG	Diclofenac	AMG	Piroxicam	AMG	Celecoxib	
Patients evaluated, *n*	32	32	49	50	85	95	
GI symptoms, *n* (%)	17 (53.1)	14 (43.8)	18 (36.7)	20 (40)	45 (52.9)	57 (60)	>0.05
Serious AEs related to the drug leading to withdrawal, *n* (%)	3 (9.4)	5 (15.6)	2 (4.1)	9 (18)	0 (0)	1 (1.1)	<0.05^1^
Cases of severe gastric and/or duodenal ulcer after treatment, *n* (%)	1 (3.1)	8 (25)	NE		4 (4.7)	10 (10.5)	<0.05
Patients with very high endoscopy score, *n* (%)	4 (12.5)	14 (43.8)	NE		21 (24.7)	21 (22.1)	<0.05

NE: not evaluated.

^1^Additionally post hoc test was performed which gave similar results.
